# Supplementary material for: A shift in transitional forests of the North American boreal will persist through 2100
Source: Commun Earth Environ. 2024 May 31;5(1):290. doi: 10.1038/s43247-024-01454-z (PMC11142915; doi:10.1038/s43247-024-01454-z)
Supplement: Supplementary file 2 — Supplemental Material [file 43247_2024_1454_MOESM2_ESM.pdf]

Title

A shift in transitional forests of the North American boreal will persist through 2100

Authors

Paul M. Montesano[1,2], Melanie Frost[1,3], Jian Li[1,3], Mark Carroll[1], Christopher S. R. Neigh[1], Matthew J. Macander[4], Joseph O. Sexton[5], Gerald V. Frost[4]

[1] NASA Goddard Space Flight Center, Greenbelt, MD, USA
[2] ADNET Systems, Inc., Bethesda, MD, USA
[3] ASRC Federal InuTeq, Beltsville, MD, USA
[4] Alaska Biological Research, Inc., Fairbanks, AK, USA
[5] TerraPulse, Inc., Potomac, MD, USA

Supplemental Information

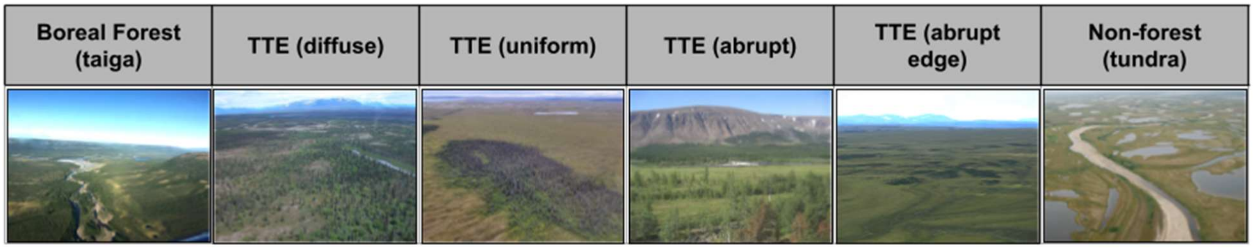

Supplementary Figure 1. Examples of various patterns in the gradient of forest structure in the North American boreal. Oblique aerial photographs of examples of the 6 of the forest gradient classes used to describe the current forest structure pattern of landscapes within the study domain.

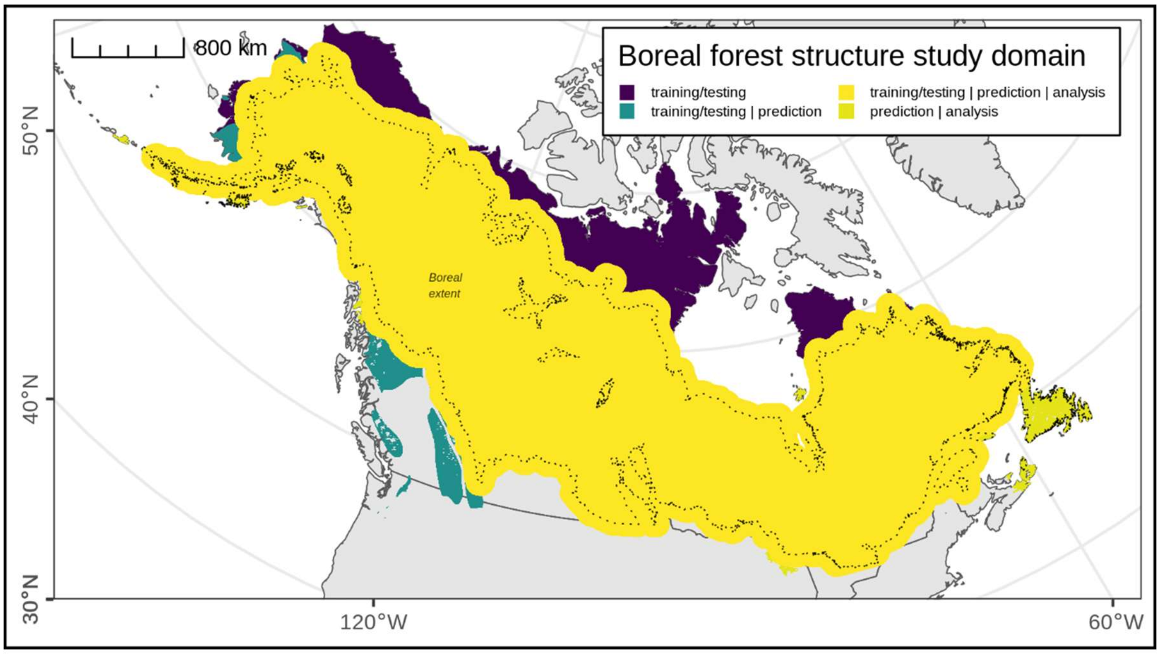

Supplementary Figure 2. The boreal forest study domain in North America. The boreal forest structure study domain is shown with 4 classes that describe the nested approach developed to perform testing and training, prediction, and analysis for model predictions of current and potential boreal forest canopy height for future time periods around a 100 km buffered boreal extent in North America, including boreal portions of islands of eastern Canada.

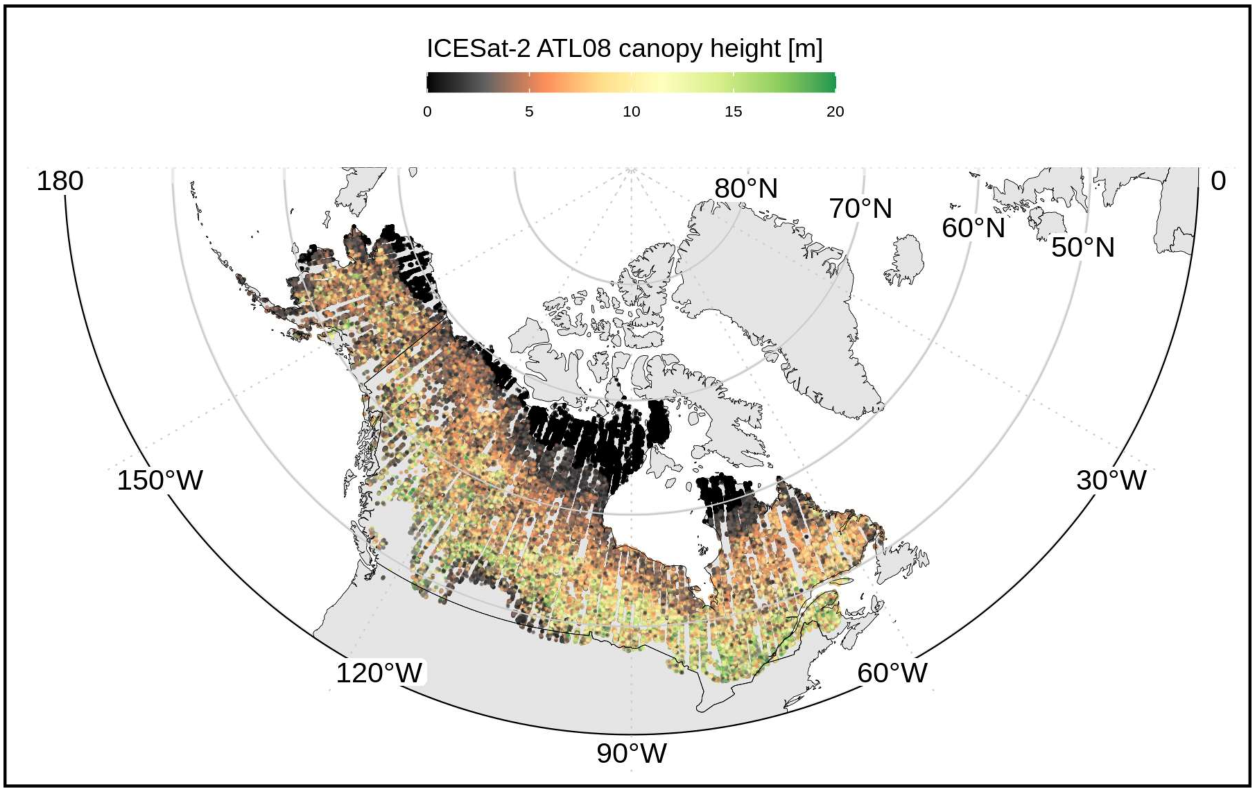

Supplementary Figure 3. Map of a sample of filtered ICESat-2 ATL08 observations of canopy height in the North American boreal. The filtered and flagged set of 20 m segment ICESat-2 ATL08 observations of canopy height (h_can_20m) used to grid the model training and testing data for predictions of current and future boreal forest canopy height. These observations include flagged heights within the *training and testing domain* that were re-coded to 0 m.

2021-2040

GCM	SSP126	SSP245	SSP370	SSP585
BCC-CSM2-MR	✓	✓	✓	✓
CanESM5	✓	✓	✓	✓
CNRM-CM6-1	✓	✓	✓	✓
GFDL-ESM4	✓		✓	
IPSL-CM6A-LR	✓		✓	✓
MIROC-ES2L	✓	✓	✓	✓
MIROC6	✓	✓	✓	✓
MRI-ESM2-0	✓	✓	✓	
CNRM-ESM2-1	✓	✓	✓	✓

2041-2060

GCM	SSP126	SSP245	SSP370	SSP585
BCC-CSM2-MR				
CanESM5	✓	✓	✓	✓
CNRM-CM6-1	✓	✓	✓	✓
GFDL-ESM4	✓		✓	
IPSL-CM6A-LR	✓	✓	✓	✓
MIROC-ES2L	✓	✓	✓	✓
MIROC6	✓	✓	✓	✓
MRI-ESM2-0	✓	✓	✓	✓
CNRM-ESM2-1	✓	✓	✓	✓

2061-2080

GCM	SSP126	SSP245	SSP370	SSP585
BCC-CSM2-MR	✓	✓	✓	✓
CanESM5	✓	✓	✓	✓
CNRM-CM6-1	✓	✓	✓	✓
GFDL-ESM4	✓		✓	
IPSL-CM6A-LR	✓	✓	✓	✓
MIROC-ES2L	✓	✓	✓	✓
MIROC6	✓	✓	✓	✓
MRI-ESM2-0	✓	✓	✓	✓
CNRM-ESM2-1	✓	✓	✓	✓

2081-2100

GCM	SSP126	SSP245	SSP370	SSP585
BCC-CSM2-MR	✓	✓	✓	✓
CanESM5	✓	✓	✓	✓
CNRM-CM6-1	✓	✓	✓	✓
GFDL-ESM4	✓		✓	
IPSL-CM6A-LR	✓	✓	✓	✓
MIROC-ES2L	✓	✓	✓	✓
MIROC6	✓	✓	✓	✓
MRI-ESM2-0	✓	✓	✓	✓
CNRM-ESM2-1	✓	✓	✓	✓

Supplementary Figure 4. **Graphical summary of the CMIP6 climate models used for ensemble canopy height prediction.** This summary shows the input availability of the gridded bioclimatic variables from 7-9 GCMs for the 4 CMIP6 SSPs across the 4 future time periods.

Supplementary Table 1. The canopy height thresholds used to filter ATL08 data according to landcover class.

Canopy Height Threshold (m)	Landcover Class
60	Closed forest, evergreen needle leaf
60	Closed forest, deciduous needle leaf
60	Closed forest, evergreen broad leaf
60	Closed forest, deciduous broad leaf
60	Closed forest, mixed
60	Closed forest, unknown
50	Open forest, evergreen needle leaf
50	Open forest, deciduous needle leaf
50	Open forest, evergreen broad leaf
50	Open forest, deciduous broad leaf
50	Open forest, mixed
50	Open forest, unknown
5	Shrubs
5	Herbaceous
5	Herbaceous Wetland
5	Moss and lichen
5	Bare/sparse vegetation

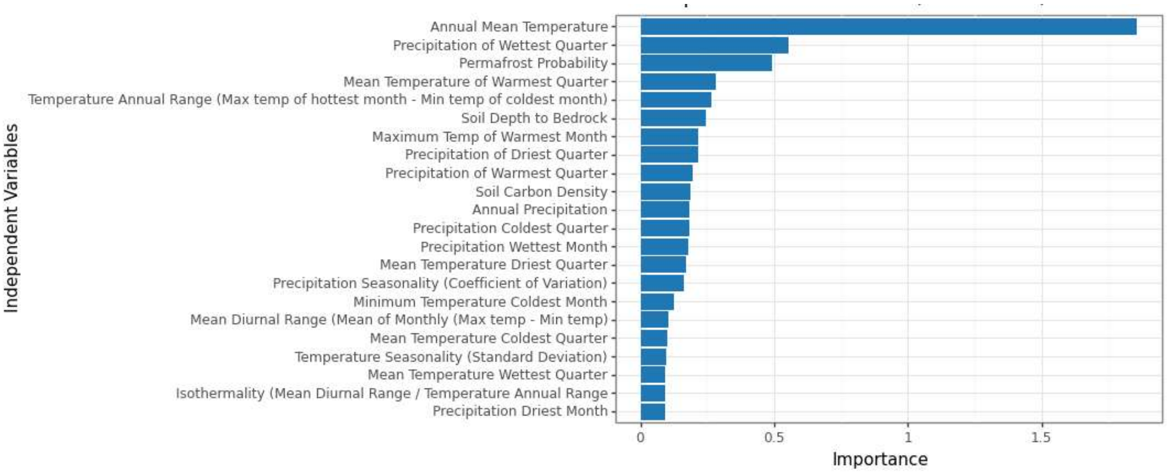

Supplementary Figure 5. **Variable importance from the canopy height prediction model.** The ranked permutation importance of the 22 predictor variables used to build a model used to predict gridded canopy height across the study domain.

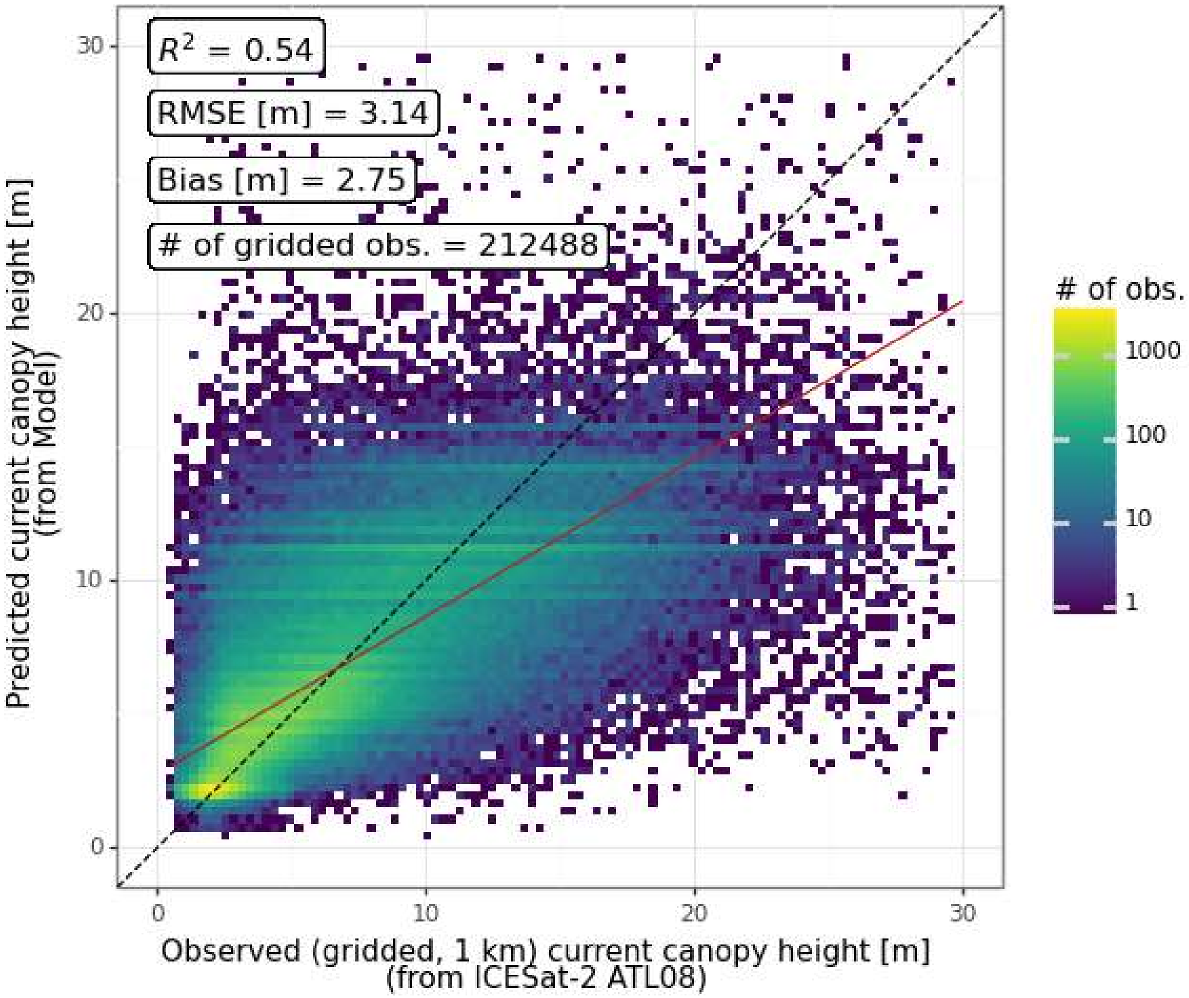

Supplementary Figure 6. The relationship of predicted and observed current (c. 2020) canopy heights in the North American boreal. Binned scatterplot of the values of the reserved testing set (n = 212,488) of predictions of current canopy height and associated gridded predictions of current ICESat-2 ATL08 observations across the study domain. The red line plots the linear relationship between the predicted and the observed values.

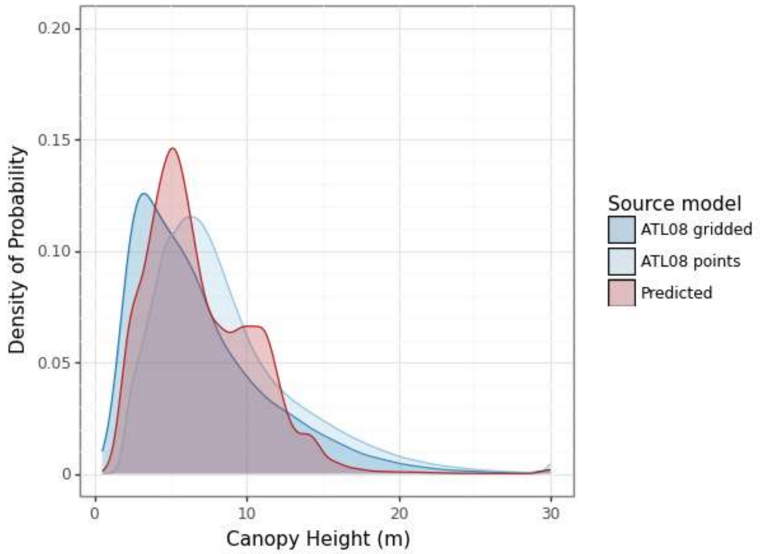

Supplementary Figure 7. Distributions of canopy heights in the North American boreal. Frequency distributions of current (c. 2020) canopy height from the reserved testing set of current predictions (red) with corresponding gridded estimates (blue) and current ICESat-2 ATL08 point observations (light blue) across the study domain feature similar distributions and medians (6.15 m, 5.82 m, 7.46 m, respectively) of canopy height.

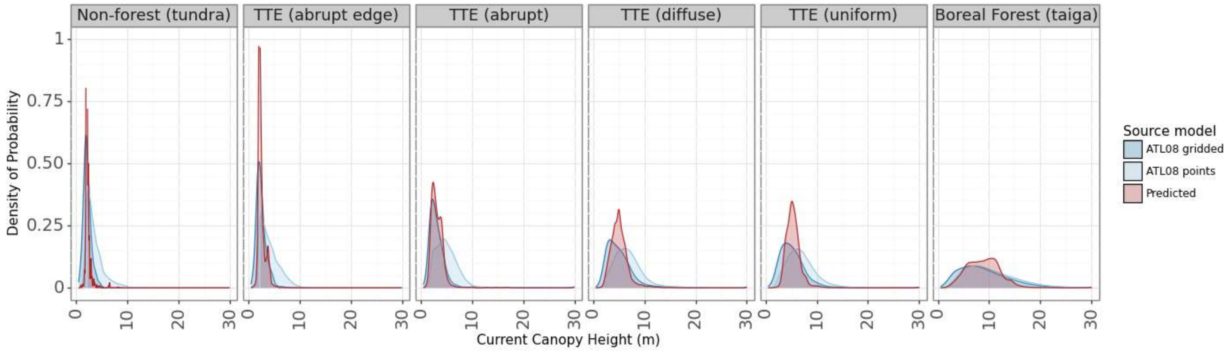

Supplementary Figure 8. Distributions of canopy heights in the North American boreal within forest gradient classes. Frequency distributions of canopy height within each landscape forest gradient class summarize the level of agreement in the reserved test set of current model predictions with corresponding grid-based ICESat-2 ATL08 observations and the full set of point-based ATL08.

**Current Canopy Height (m)** median +/- nmad	Non-forest (tundra)	TTE (abrupt edge)	TTE (abrupt)	TTE (diffuse)	TTE (uniform)	Boreal Forest (taiga)	Total Study Domain
point-based ATL08	3.1 +/- 1.2	3.7 +/- 1.7	4.8 +/- 2.1	6.5 +/- 2.5	6.6 +/- 2.6	9.4 +/- 4.8	7.6 +/- 3.8
grid-based ATL08	2.1 +/- 0.6	2.3 +/- 0.8	2.8 +/- 1.2	4.6 +/- 2.2	4.9 +/- 2.2	8.2 +/- 4.6	5.8 +/- 3.8
Model Predicted	2.2 +/- 0.1	2.3 +/- 0.4	3 +/- 1.1	5 +/- 1.3	5.2 +/- 1.1	8.9 +/- 3.4	6.2 +/- 3.3

Supplementary Table 2. The summaries for each landscape forest gradient class of the three distributions of current canopy heights. The median and normalized median absolute deviation from the distributions of current canopy heights within each landscape forest gradient class, as well as for the total study domain. These distribution parameters summarize the level of agreement in the reserved test set of current (c. 2020) canopy height from the corresponding reference observations (point-based and grid-based ICESat-2 ATL08) with model predictions.

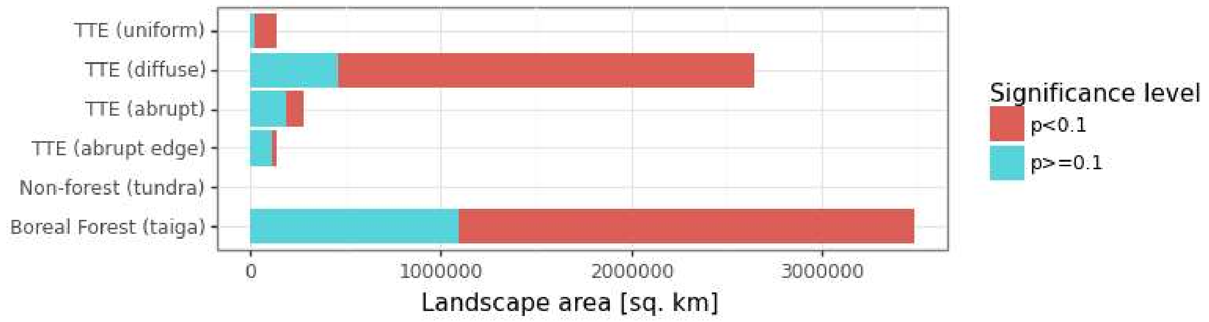

Supplementary Figure 9. The significance of the multi-decadal trend in Landsat-derived tree canopy cover at the hydrobasin (landscape) level. Results summarize, by landscape forest gradient class, the proportion of landscapes whose median p-value is less than 0.1. Most landscapes have median values less than this threshold, suggesting a preponderance of significant trends at mesoscales.

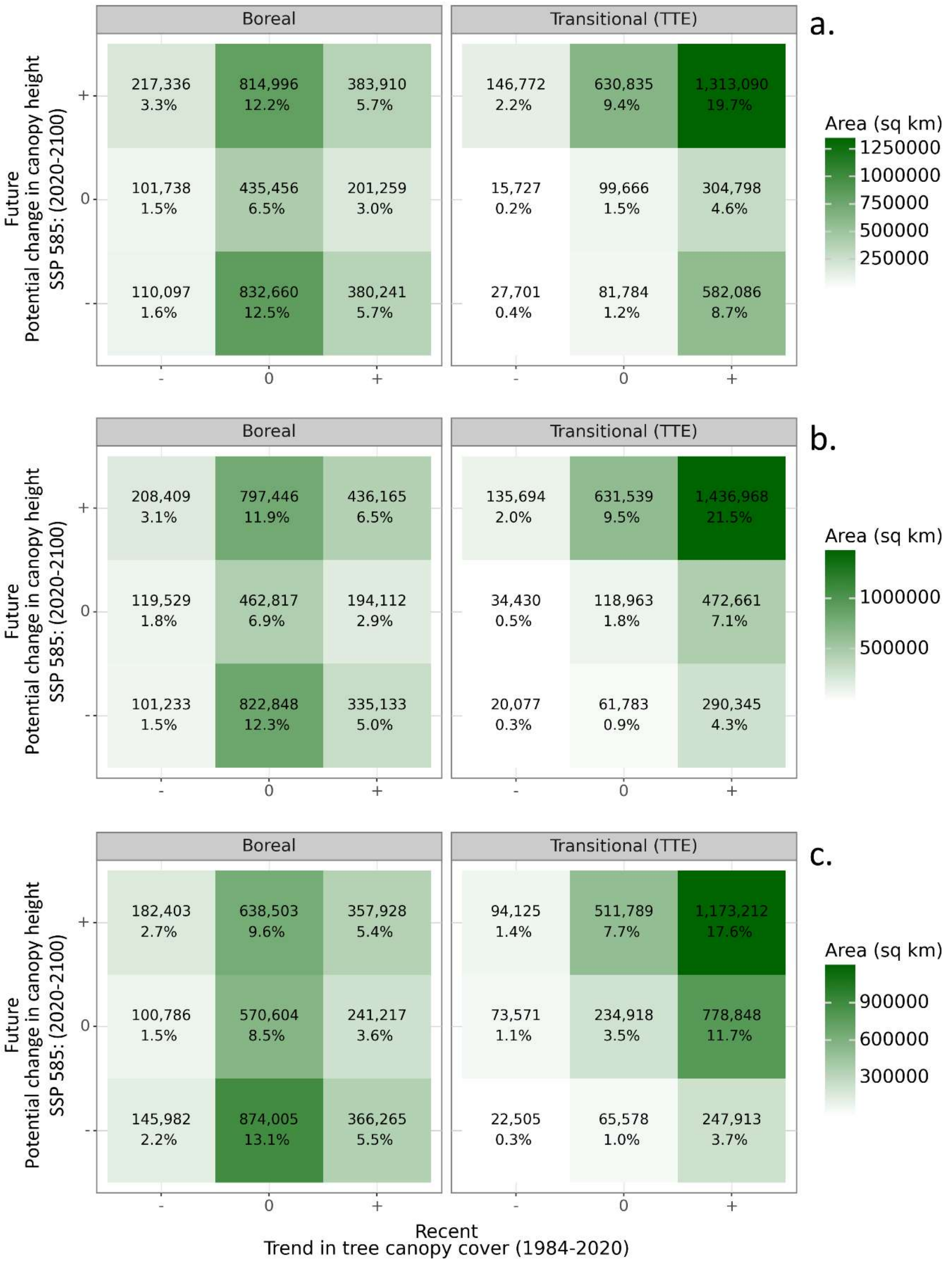
Boreal
Transitional (TTE)
a.
b.
c.
Future
Potential change in canopy height
SSP 585: (2020-2100)
Recent
Trend in tree canopy cover (1984-2020)
Area (sq km)
1250000
1000000
750000
500000
250000
217,336 3.3%
814,996 12.2%
383,910 5.7%
146,772 2.2%
630,835 9.4%
1,313,090 19.7%
101,738 1.5%
435,456 6.5%
201,259 3.0%
15,727 0.2%
99,666 1.5%
304,798 4.6%
110,097 1.6%
832,660 12.5%
380,241 5.7%
27,701 0.4%
81,784 1.2%
582,086 8.7%
208,409 3.1%
797,446 11.9%
436,165 6.5%
135,694 2.0%
631,539 9.5%
1,436,968 21.5%
119,529 1.8%
462,817 6.9%
194,112 2.9%
34,430 0.5%
118,963 1.8%
472,661 7.1%
101,233 1.5%
822,848 12.3%
335,133 5.0%
20,077 0.3%
61,783 0.9%
290,345 4.3%
182,403 2.7%
638,503 9.6%
357,928 5.4%
94,125 1.4%
511,789 7.7%
1,173,212 17.6%
100,786 1.5%
570,604 8.5%
241,217 3.6%
73,571 1.1%
234,918 3.5%
778,848 11.7%
145,982 2.2%
874,005 13.1%
366,265 5.5%
22,505 0.3%
65,578 1.0%
247,913 3.7%
900000
600000
300000

Supplementary Figure 10. A landscape-scale area summation and classification of the direction of structural changes from future (2020-2100) and recent (1984-2020) time periods across the North American boreal forest and transitional landscapes of the TTE for 3 CMIP6 scenarios. Changes are classified based on median values captured across individual landscapes as negative (decreasing; -), no change (stable; 0), or positive (increasing; +). Recent changes are from observations of 1984-2020 tree canopy cover trends and classes of future changes are from predictions of future (2100) potential canopy height changes, assuming SSP245, from current conditions. Numbers (top) represent the total area of each class and (bottom) the proportion of each class’s area relative to the entire North American study domain. Results shown for a SSP 585, b SSP 370, and c SSP 126.
